# hUCMSC-derived extracellular vesicles relieve cisplatin-induced granulosa cell apoptosis in mice by transferring anti-apoptotic miRNAs

**DOI:** 10.7555/JBR.37.20230310

**Published:** 2024-05-29

**Authors:** Wenjing Tang, Haiyan Yan, Xiaojun Chen, Yanan Pu, Xin Qi, Liyang Dong, Chuan Su

**Affiliations:** 1 State Key Laboratory of Reproductive Medicine and Offspring Health, Nanjing Medical University, Nanjing, Jiangsu 211166, China; 2 Department of Pathogen Biology & Immunology, Nanjing Medical University, Nanjing, Jiangsu 211166, China; 3 Department of Nuclear Medicine, the Affiliated Hospital of Jiangsu University, Zhenjiang, Jiangsu 212000, China

**Keywords:** chemotherapy, fertility preservation, extracellular vesicles, granulosa cell, apoptosis, microRNAs

## Abstract

Premature ovarian insufficiency (POI) caused by chemotherapy is a common complication in female cancer survivors of childbearing age. Traditional methods, including mesenchymal stem cell (MSC) transplant and hormone replacement therapy, have limited clinical application because of their drawbacks, and more methods need to be developed. In the current study, the potential effects and underlying mechanisms of human umbilical cord MSC-derived extracellular vesicles (hUCMSC-EVs) were investigated in a cisplatin (CDDP)-induced POI mouse model and a human granulosa cell (GC) line. The results showed that hUCMSC-EVs significantly attenuated body weight loss, ovarian weight loss, ovary atrophy, and follicle loss in moderate-dose (1.5 mg/kg) CDDP-induced POI mice, similar to the effects observed with hUCMSCs. We further found that the hUCMSC-EVs inhibited CDDP-induced ovarian GC apoptosis by upregulating anti-apoptotic miRNA levels in GCs, thereby downregulating the mRNA levels of multiple pro-apoptotic genes. In general, our findings indicate that the moderate-dose chemotherapy may be a better choice for clinical oncotherapy, considering effective rescue of the oncotherapy-induced ovarian damage with hUCMSC-EVs. Additionally, multiple miRNAs in hUCMSC-EVs may potentially be used to inhibit the chemotherapy-induced ovarian GC apoptosis, thereby restoring ovarian function and improving the life quality of female cancer patients.

## Introduction

Premature ovarian insufficiency (POI) is characterized as the degeneration or complete loss of ovarian function in women younger than 40 years old and is not an extremely rare disorder. This disorder is characterized by elevated gonadotropin levels, decreased estradiol levels, amenorrhea (for most), and significantly reduced fertility^[1]^. POI is diagnosed based on biochemical criteria, which include serum follicular stimulating hormone levels exceeding 25 mIU/L and estradiol levels below 50 pmol/L in women who have experienced 12 consecutive months without menstruation^[2]^. The incidence of POI in women increases with age: POI affects 1%–2% of women under 40 years old, and the incidence increases to 10% for women under 45 years old^[1]^. A significant issue associated with POI is compromised reproductive health, potentially leading to infertility in women of reproductive age. The impaired fertility often stems from defects in the formation of the primordial follicle pool, as well as difficulties in follicular recruitment, maturation, and premature follicular atresia^[3]^. Additionally, POI significantly disrupts daily life for the patients, manifesting in symptoms like hot flashes, sweating, sleep disturbances, vaginal dryness, and mental disorders like depression^[4]^. Therefore, protecting or restoring ovarian function is crucial for fertility preservation and the life quality of adolescent and young female cancer survivors.

POI is categorized into two types: primary and secondary. Primary POI may result from factors, such as genetic predisposition, autoimmune responses, inflammation, and metabolic disorders, while secondary POI is generally caused by iatrogenic factors, such as chemotherapy, radiotherapy, and surgery^[3]^. Approximately 88.8% of patients aged under 45 years who receive modern cancer treatments survive more than five years after diagnosis^[5]^. As the first-line agent for nearly 30 common carcinomas, *cis*-diammine dichloro platinum (Ⅱ) (CDDP), also known as cisplatin, and its derivatives induce DNA damage and trigger cell apoptosis in the fast-proliferating cancer cells^[6]^. However, CDDP is also toxic to normal cells that divide at a fast rate^[6]^. In the ovaries, granulosa cells (GCs) proliferate quickly to promote ovarian follicle development, and GCs in small follicles are more proliferative than those in large follicles^[7]^. CDDP impairs ovarian function and increases the POI morbidity by over 10% in female cancer survivors of reproductive age^[5]^. Concurrently, studies have indicated that injury and apoptosis of ovarian GCs play a critical role in the etiology of POI, especially the GC apoptosis induced by CDDP chemotherapy^[8]^. Therefore, inhibiting GC apoptosis may be a prevention strategy for treating chemotherapy-induced POI.

GCs are involved in the conversion of androgens to estrogens and the synthesis of progesterone. A reduction in the GC number leads to decreased levels of endogenous estrogen and progesterone, as well as disruption of the hypothalamic-pituitary-ovarian axis, resulting in menopausal-like symptoms^[9]^. Additionally, the regulation of follicle development and fate determination is mediated by estrogen, anti-Müllerian hormone, and other proteins produced by GCs. Consequently, an increase in GC apoptosis triggers the process of follicular atresia^[10]^.

Mesenchymal stem cells (MSCs), known for their roles in regulating immune activities, preserving organ homeostasis, and regenerating injured tissues, are multipotent stem cells widely used in cell-based therapies^[11]^. Compared with other types of MSCs, human umbilical cord-derived MSCs (hUCMSCs) offer several advantages, because of the simplicity of their harvest, separation, culture, and purification processes. Additionally, hUCMSCs retain their stemness after multiple passages and expansion. Moreover, the surface antigens of hUCMSC are minimally expressed, causing insignificant rejection reactions and lenient matching requirements^[12]^. These features have facilitated the widespread use of hUCMSCs in allografts. Nevertheless, the practical application of MSC allografts faces several challenges, including storage and transportation difficulties, as well as safety concerns such as the risk of rejection reactions and tumorigenicity^[13]^. In contrast, extracellular vesicles (EVs) originating from MSCs offer a more biologically stable alternative because of their reduced immunogenicity^[13]^. EVs are nanoscale lipid-bound complexes containing proteins, lipids, signaling molecules, and nucleic acids, including an abundance of small non-coding RNAs^[14]^. EVs are now considered an additional mechanism for intercellular communication and material exchange. The transplanted MSCs exert their therapeutic effects mainly through paracrine mechanisms rather than direct cell replacement, with EVs being indispensable components in MSC paracrine secretion^[15]^. However, the role of hUCMSC-EVs in restoring ovarian function after chemotherapy has not been fully investigated.

In the current study, we evaluated the potential of hUCMSC-EVs in mitigating the adverse effects on ovaries caused by CDDP-based chemotherapy.

## Materials and methods

### Cell culture

The hUCMSCs (Cat. #PCS-500-010) and human GC line KGN (Cat. #ZY-H343) were purchased from ATCC (Manassas, VA, USA). hUCMSCs were grown in OriCell^TM^ Medium for hUCMSCs (Cat. # HUXUC-90062, Cyagen, Guangzhou, Guangdong, China), while KGN cells were grown in low-glucose DMEM (Cat. #11885084, Gibco, Carlsbad, CA, USA). These cells were all supplemented with 10% fetal bovine serum (Cat. #10099158, Gibco) and 1% penicillin-streptomycin (Cat. #15070063, Gibco), and cultured under 5% CO_2_ at 37 ℃.

### Preparation of hUCMSC-EVs

hUCMSCs were cultured in 10-cm culture dishes until reaching approximately 80% confluence. Following two washes with Dulbecco's Phosphate Buffered Saline (DPBS), the culture medium was substituted with serum & EV-free medium (Cat. #abs9774, Absin, Shanghai, China). After 24 h, the culture supernatant was collected and subjected to centrifugation at 300 *g* for 10 min, followed by 2000 *g* for 20 min to eliminate dead cells. Subsequently, the supernatant was filtered through 0.45-μm filters to remove cell debris. The filtered supernatant underwent ultracentrifugation (125000 *g*; Beckman Coulter XPN-100, Brea, CA, USA) at 4 ℃ for three hours. After discarding the supernatant, the pellets were collected and resuspended in DPBS. The particle size distribution of hUCMSC-derived EVs was assessed using a Zetasizer Nano ZS (Malvern Instruments Ltd., Worcestershire, UK), and the quantification of hUCMSC-EVs was carried out with a BCA protein assay kit (Cat. #P0010, Beyotime, Shanghai, China). The nanoparticle trafficking analysis indicated that 200 μg of hUCMSC-derived EVs contained about 6 × 10^9^ EV particles under our experimental conditions. The resuspended hUCMSC-EVs were stored at −80 ℃ until needed.

### Transmission electron microscopy (TEM)

Purified hUCMSC-EVs were treated with 4% paraformaldehyde and 4% glutaraldehyde at room temperature. The fixed hUCMSC-EVs were deposited onto a carbon-coated copper grid, which was subsequently immersed in a 2% phosphotungstic acid solution (pH 7.0) for 30 s. The grid was then examined using a transmission electron microscope (JEM-1200EX, JEOL, Tokyo, Japan).

### Protein extraction from extracellular vesicles and Western blotting analysis

Total protein from extracellular vesicles was extracted using an 8 mol/L urea buffer (composed of 50 mmol/L Tris-HCl pH 8.2, 75 mmol/L NaCl, and 8 mol/L urea) supplemented with a protease inhibitor cocktail (Cat. #B14001, Bimake, Houston, TX, USA). The protein concentration was determined using a Bradford protein assay kit (Cat. #P0006, Beyotime). Equal amounts of proteins (15 μg) were separated by electrophoresis on 10% SDS-PAGE gels and then transferred onto polyvinylidene difluoride membranes (Cat. #1620177, Bio-Rad, Hercules, CA, USA). After blocking with 5% non-fat milk at room temperature for two hours, the membranes were incubated with diluted primary antibodies overnight at 4 ℃. The following primary antibodies were used: anti-HSP70 (Cat. #4872T, Cell Signaling Technology, Boston, MA, USA, 1∶1000) and anti-TSG101 (Cat. #28283-1-AP, Proteintech, Wuhan, Hubei, China, 1∶5000). Subsequently, the membranes were washed with TBST and incubated with HRP-conjugated secondary antibodies at room temperature for two hours. Finally, the signals were visualized through chemiluminescence using ChemiDoc XRS (Bio-Rad).

### Establishment of the CDDP-induced POI mouse model

The approval for all animal experiments was obtained from the Institutional Animal Care and Use Committees (IACUC) of Nanjing Medical University, Nanjing, China (Approval No. 2109046). All the animal experiments were carried out following the guidelines for the care and use of animals established by IACUC. Female BALB/c mice aged eight weeks were procured from the Laboratory Animal Center of Nanjing Medical University and housed in a specific-pathogen-free animal facility with a 12-hour light/dark cycle.

To establish a chemotherapy-induced POI model, mice were intraperitoneally injected with *cis*-diaminodichloroplatinum (CDDP; Cat. #HY-17394, MedChemExpress, Monmouth Junction, NJ, USA) at a dose of 1.5 mg/kg body weight for seven consecutive days.

### hUCMSC and hUCMSC-EVs transplantation

BALB/c mice were randomly assigned to four groups: the normal group (normal mice without any treatment, *n* = 4), the CDDP-induced POI group (POI mice, *n* = 4), the hUCMSC-treated group (POI mice injected with 2 × 10^6^ hUCMSCs twice a week *via* the tail vein, *n* = 4), and the hUCMSC-EV-treated group (POI mice injected with 200 μg hUCMSC-EVs twice a week *via* the tail vein, *n* = 4). At 7 or 21 days post-initiation of the rescue protocol, three mice from each group were sacrificed, and their GC and ovary samples were isolated for subsequent experiments.

### *In vivo* spectral imaging

We mixed 1,1′-dioctadecyl-3,3,3′,3′-tetramethylindotricarbocyanine iodide (DiR, 5 pg/L; Cat. #D12731, Invitrogen, Carlsbad, CA, USA) with hUCMSC-EVs and incubated the mixture at 37 ℃ for 30 min. Then, the DiR-labeled EVs were washed three times with DPBS by ultracentrifugation at 120000 *g* for two hours. Then, 3 × 10^9^ DiR-labeled hUCMSC-EVs were injected into BALB/c mice through the tail vein (seven days after CDDP treatment), and the fluorescence intensity was detected with IVIS Spectrum (PerkinElmer, Waltham, MA, USA) to indicate the distribution of hUCMSC-EVs. The instrument conducted *in vivo* spectral imaging at wavelengths of 690–850 nm, with an exposure time of 150 ms per image frame.

### GCs isolation

Bilateral ovaries of the mice were gathered in M2 medium (Cat. #M1250, EasyCheck, Nanjing, Jiangsu, China) and finely minced. Then, oocytes were removed with a capillary under a stereomicroscope (Nikon, Tokyo, Japan), and tissue fragments were removed by passing through a 200-mesh nylon membrane. After centrifuging the liquid at 1000 *g* for five minutes, we discarded the supernatant, and washed the deposit twice to acquire the isolated GCs.

### Paraffin embedding and hematoxylin and eosin (H&E) staining

Ovarian tissues were fixed in 4% paraformaldehyde at room temperature for 48 h, and were subsequently dehydrated in a series of ethanol concentrations (70%, 80%, 90%, and 100%). Following dehydration, the tissues were immersed in a solution of xylene and ethanol (1∶1) and then in 100% xylene. The ovarian tissues were embedded in paraffin and sectioned into 5-µm thick slides. The H&E staining was carried out using a hematoxylin staining solution (Cat. #G1004, Servicebio, Wuhan, Hubei, China) and an eosin staining solution (Cat. #E607321, Sangon Biotech, Shanghai, China).

### *In vivo* and *in vitro* apoptosis assay

GCs undergoing apoptosis were identified using a TUNEL assay performed with an *In Situ* Cell Death Detection Kit (Cat. #11684795910, Roche, Basel, Switzerland) according to the manufacturer's instructions. 3,3′-Diaminobenzidine served as the substrate for visualization, and the nuclei were counterstained with a hematoxylin staining solution.

KGN cells undergoing apoptosis were assessed by staining with Annexin V-FITC (Cat. #556420, BD Biosciences, Franklin Lakes, NJ, USA) following a previous authoritative study^[16]^ and the manufacturer's protocol. The percentage of apoptotic cells was determined using a FACS Verse flow cytometer (BD Biosciences), acquiring at least 100000 events. The obtained results were analyzed using FlowJo software (Tree Star v10.8.1).

### Total RNA isolation and quantitative reverse transcription-PCR

hUCMSC-EVs and cells were homogenized in TRIzol^TM^ Reagent (Cat. #15596026, Invitrogen), and RNA was extracted as described by the manufacturer. Total RNA (1 μg) was reverse transcribed with 5× All-In-One MasterMix (Cat. #G592, Applied Biological Materials, Richmond, BC, Canada). Equal miRNAs were reverse transcribed with a miRNA All-In-One cDNA Synthesis Kit (Cat. #G898, Applied Biological Materials). Then, we amplified the products with Blastaq Green 2× qPCR MasterMix (Cat. #G895, Applied Biological Materials) on the Q5 Real-Time PCR System (Applied Biosystems, Waltham, MA, USA). The relative expression levels of mRNA and miRNA were assessed using the 2^−ΔΔCt^ method, and the normalization was performed with 18S rRNA primers for mRNAs and U6 snRNA primers (Cat. #MPH00001, Applied Biological Materials) for miRNAs, respectively. All primer sequences are listed in ***Table 1***. The specificity of PCR products was assessed by melting curve analyses.

**Table 1:** The primers for mouse genes and human miRNAs

Genes/miRNAs	Forward primers (5′-3′)	Reverse primers (5′-3′)
*Itm2b*	TGTGCACGACTTCAACAAGAAACT	GTAGGACTGAGGCAGGTAGGT
*Slc30a8*	CATGCCCTGGGGGATGTATT	AGGCCCTTTGGAACACCTTC
*Arid3b*	ATCAACGGCAGGGAAGACAG	CTGGTTTCTGGGCAAACAGC
*Nr6a1*	GAGGCCGGAACAAGAGCATT	TTCCACAGACCTACTGGATGA
*Thrsp*	AGGTGACGCGGAAATACCAG	TCTCTCGTGTAAAGCGATCTTCAG
*Usp44*	CTAAAGGCCAATTCGGCGAC	CGGGAGCATCTGAAAACTGG
*Crebzf*	TTCCAGCAGTCCAAAGATGT	CCCATTCATGCTCAAGTGCAG
*Cdkn1b*	CAGACGTAAACAGCTCCGAATTA	GGCAGATGGTTTAAGAGTGCC
*Gabra1*	ATGTTCTAGCAGGGAAGCGAG	GAGGGCTGTCCATAGCTTCTTC
*18S*	CGGACAGGATTGACAGATTGATAG	ATGCCAGAGTCTCGTTCGTTAT
has-miR-100-5p	AACCCGTAGATCCGAACTTGTG	–
has-miR-21-5p	TAGCTTATCAGACTGATGTTGA	–
has-miR-143-3p	TGAGATGAAGCACTGTAGCTC	–
has-miR-125b-5p	TCCCTGAGACCCTAACTTGTGA	–
has-miR-26a-5p	TTCAAGTAATCCAGGATAGGCT	–
has-let-7i-5p	TGAGGTAGTAGTTTGTGCTGTT	–
has-miR-221-3p	AGCTACATTGTCTGCTGGGTTTC	–
has-miR-191-5p	CAACGGAATCCCAAAAGCAGCTG	–
has-miR-222-3p	AGCTACATCTGGCTACTGGGT	–

### Statistical analysis

Statistical analyses were performed using GraphPad Prism (Version 8.0). The data were presented as mean ± standard error of the mean. The normal distribution of the data was confirmed by the Kolmogorov–Smirnov test. One-way ANOVA followed by a Bonferroni post hoc test was used for two-group comparison among three or more groups. The differences at *P* < 0.05 were considered statistically significant.

## Results

### Identification of extracellular vesicles derived from hUCMSC

**Figure 1:**
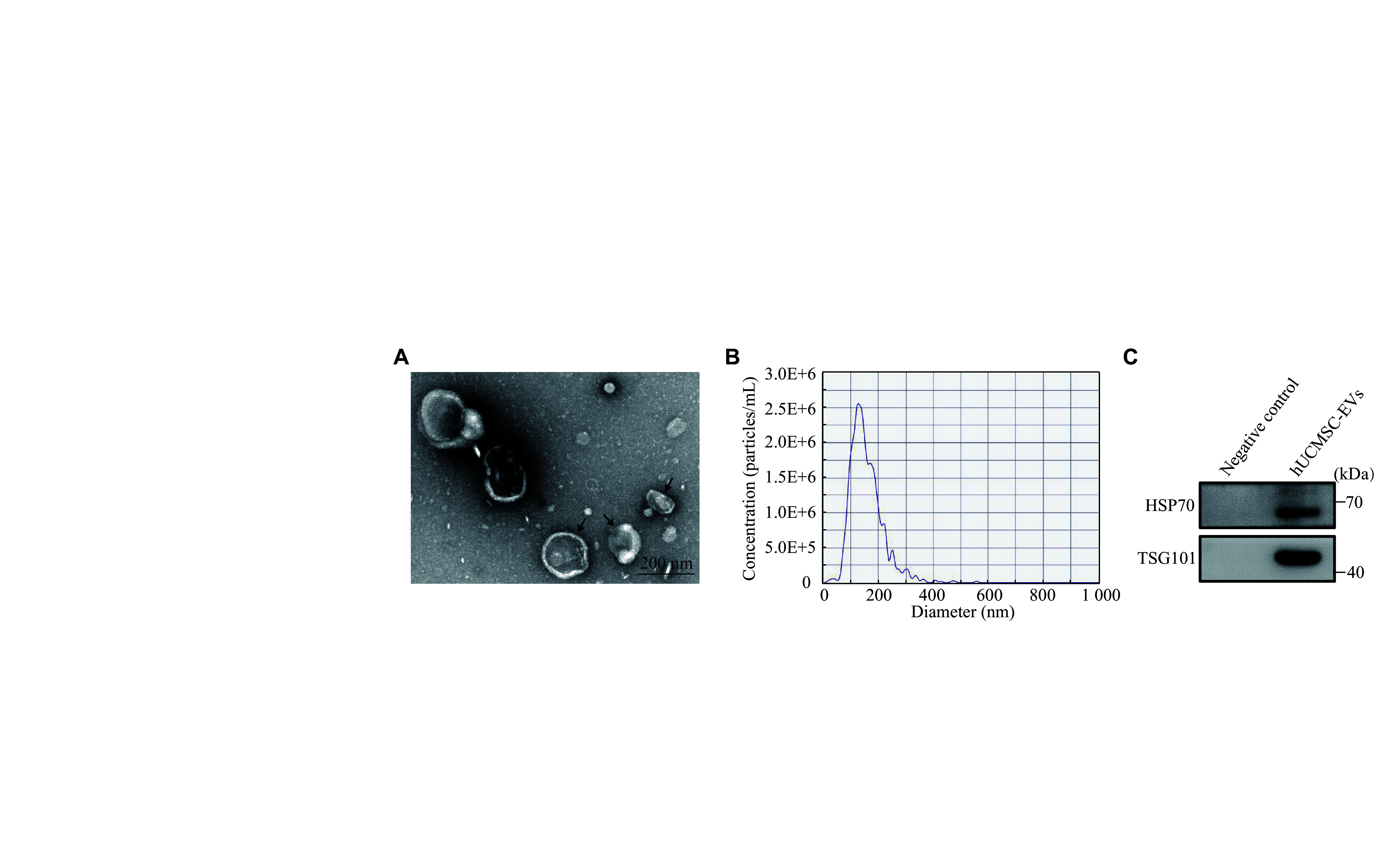
Characterization of human umbilical cord mesenchymal stem cell-derived extracellular vesicles (hUCMSC-EVs). A: The size and morphology of hUCMSC-EVs observed by transmission electron microscopy. Arrows indicate typical hUCMSC-EVs. Scale bar, 200 nm. B: Nanoparticle tracking analysis showed the size distribution of hUCMSC-EVs. C: The canonical EV markers HSP70 and TSG101 in hUCMSC-EVs were detected by Western blotting. Serum & EV-free medium served as the negative control sample. Abbreviations: hUCMSC-EVs, human umbilical cord-derived mesenchymal stem cell-derived extracellular vesicles; HSP70, heat shock 70 kDa protein; TSG101, tumor susceptibility gene 101 protein.

hUCMSCs were stem cells isolated from the umbilical cord interstitium of newborns^[17]^. For the hUCMSCs purchased and used in the laboratory, we have already confirmed their adipogenic, osteogenic, and chondrogenic differentiation capabilities in our previous study^[18]^. EVs were extracted from hUCMSCs, and their characteristics were identified through TEM imaging, nanoparticle trafficking analysis, and Western blotting. The TEM imaging revealed that hUCMSC-EVs were spheres exhibiting a dish-like morphology with a complete membrane structure (***Fig. 1A***). The diameters of most hUCMSC-EVs ranged from 100 nm to 200 nm, with a size distribution ranging from 30 nm to 550 nm (***Fig. 1B***). The result of Western blotting confirmed that the EV surface markers HSP70 and TSG101 were positively expressed in proteins extracted from hUCMSC-EVs (***Fig. 1C***). These findings indicated a successful harvest of hUCMSC-EVs from the hUCMSC culture medium.

### hUCMSC-EVs reversed the weight loss and ovary atrophy in moderate-dose CDDP-induced POI mice

**Figure 2:**
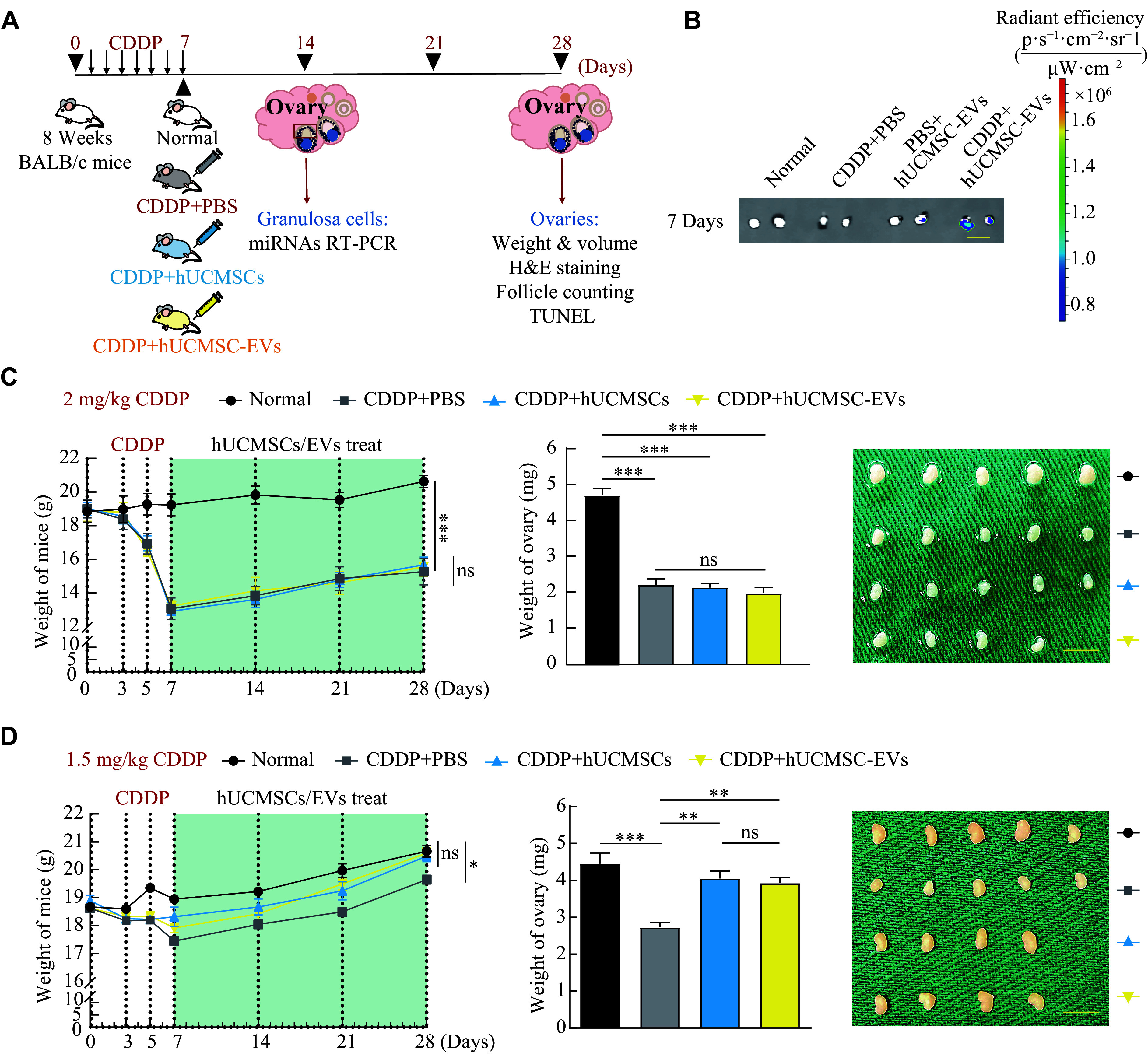
hUCMSCs and hUCMSC-EVs transplantation attenuated the CDDP-induced weight loss and ovary atrophy in mice. A: The schematic diagram of the experimental procedure used to establish a CDDP-induced POI mouse model to investigate the restoration effects of hUCMSCs and hUCMSC-EVs. The mice were intraperitoneally injected with CDDP for 7 consecutive days, followed by administration of 2 × 10^6^ hUCMSCs or 200 μg hUCMSC-EVs for 21 days twice a week (*n* = 4 for each group). B: The enrichment of DiR-labeled hUCMSC-EVs in CDDP-injured ovaries (2 mg/kg CDDP) after tail vein administration for 7 days. Scale bar, 0.5 cm. C and D: Comparison of body weight (left; *n* = 4), ovarian weight (middle; *n* = 4), and macroscopic ovarian size (right) of the control mice and the high dose (2 mg/kg body weight; C) or moderate dose (1.5 mg/kg body weight; D) CDDP-induced POI mice treated with or without hUCMSCs and hUCMSC-EVs on the 28th day. Scale bar, 0.5 cm. Data are presented as mean ± standard error of the mean. Statistical analyses were performed by one-way ANOVA followed by a Bonferroni post hoc test for four-group comparisons. ^*^P < 0.05, ^**^P < 0.01, and ^***^P < 0.001. Abbreviations: CDDP, *cis*-diammine dichloro platinum (Ⅱ); hUCMSCs, human umbilical cord-derived mesenchymal stem cells; EVs, extracellular vesicles; DiR, 1,1′-dioctadecyl-3,3,3′,3′-tetramethylindotricarbocyanine iodide; ns, not significant.

The intraperitoneal injection of mice with CDDP for seven consecutive days is a widely used protocol to construct a chemotherapy-induced POI mouse model^[19–20]^. For female cancer patients, the CDDP dose used in chemotherapy is usually in the range of 1.33 to 2.67 mg/kg body weight^[21]^. Therefore, we constructed two POI mouse models induced by high-dose CDDP (2 mg/kg body weight) and moderate-dose CDDP (1.5 mg/kg body weight). To evaluate the therapeutic effects of hUCMSCs and hUCMSC-EVs on the POI mice, especially their effects on ovarian function, we transplanted hUCMSCs or hUCMSC-EVs into the POI mice on the seventh day post the initiation of CDDP administration, and the treatment lasted for three weeks. On the 14th and 28th days, GCs and ovaries were harvested (***Fig. 2A***).

We injected DiR-labeled hUCMSC-EVs into POI mice *via* the tail vein and observed intense signals even in the moderate-dose CDDP-injured ovaries. In contrast, very few DiR signals were gathered in the control ovaries (***Fig. 2B***). This finding indicates that hUCMSC-EVs may target injured ovaries and play a specific role in injured ovaries.

The transplantation of hUCMSCs or hUCMSC-EVs failed to reverse the reduction in body weight and ovarian mass observed in the POI mouse model that received the high-dose CDDP treatment, and did not attenuate ovary atrophy in these mice (***Fig. 2C***). However, in the moderate-dose CDDP-induced POI mice, their body weight and ovarian weight exhibited a significant restoration following an equal transplantation of hUCMSC or hUCMSC-EV on the 28th day, and the ovary atrophy was also significantly attenuated (***Fig. 2D***). These results indicate that hUCMSC or hUCMSC-EVs transplant therapy may attenuate weight loss and ovarian atrophy induced by the moderate- but not high-dose CDDP-induced POI.

### hUCMSC-EVs reversed the follicle loss in the moderate-dose CDDP-induced POI mice by inhibiting GC apoptosis

**Figure 3:**
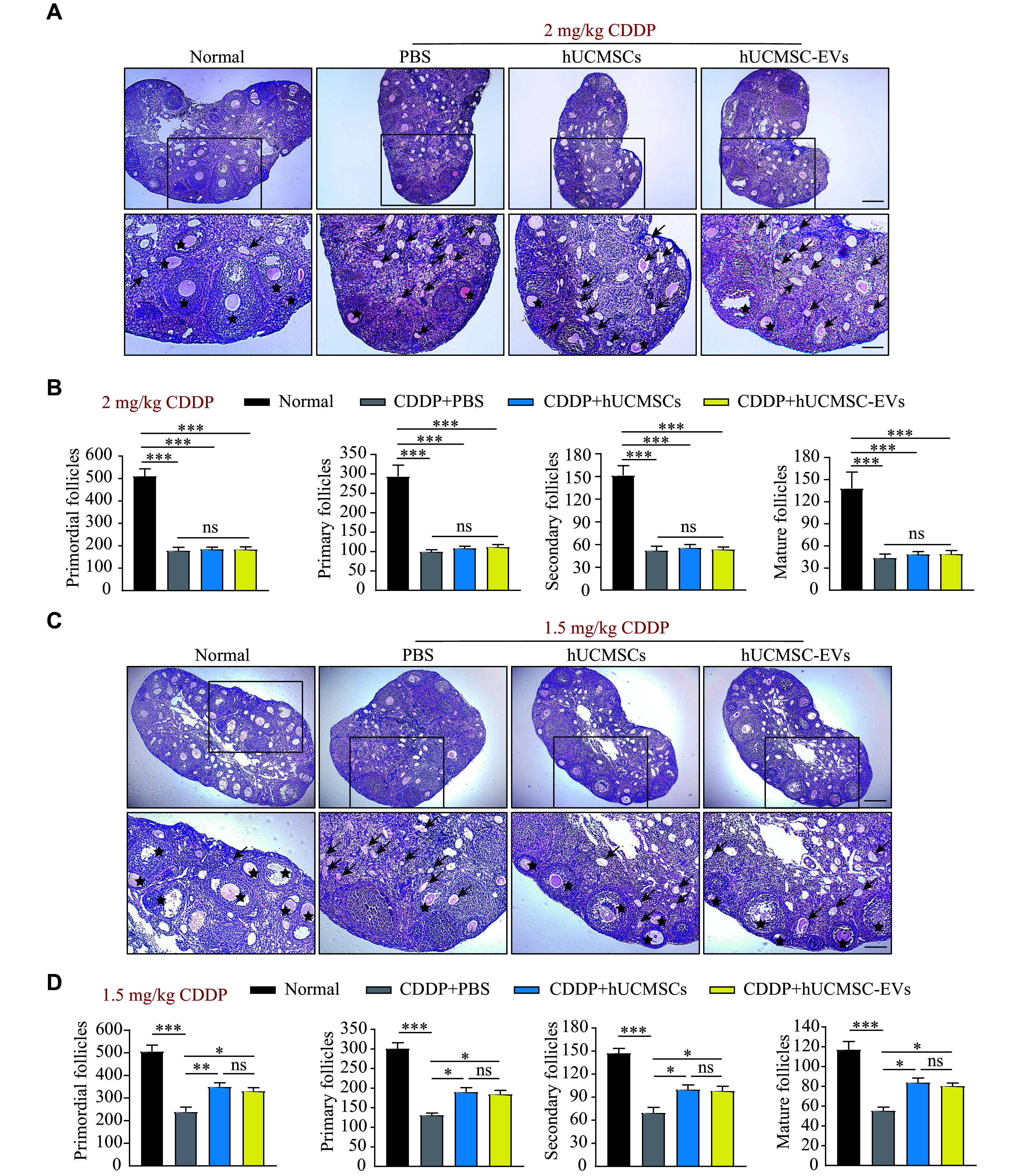
hUCMSCs and hUCMSC-EVs rescued the follicle loss at all stages in the POI mouse model. Mice were intraperitoneally injected with CDDP for seven consecutive days, followed by administration of 2 × 10^6^ hUCMSCs or 200 μg hUCMSC-EVs for 21 days twice a week (*n* = 4 for each group). A and B: The morphology of the ovary (A) and the number of follicles at different stages (B) in control mice and the high-dose (2 mg/kg body weight) CDDP-induced premature ovarian insufficiency mice treated with or without hUCMSCs and hUCMSC-EVs on the 28th day. Stars indicate normal follicles and arrows indicate atretic follicles. Scale bars, 200 μm (upper) and 100 μm (lower). C and D: The morphology of the ovary (C) and the number of follicles at different stages (D) in control mice and the moderate-dose (1.5 mg/kg body weight) CDDP-induced POI mice treated with or without hUCMSCs and hUCMSC-EVs on the 28th day. Stars indicate normal follicles and arrows indicate atretic follicles. Scale bars, 200 μm (upper) and 100 μm (lower). Data are presented as mean ± standard error of the mean. Statistical analyses were performed by one-way ANOVA followed by a Bonferroni post hoc test for four-group comparisons. ^*^P < 0.05, ^**^P < 0.01, and ^***^P < 0.001. Abbreviations: CDDP, *cis*-diammine dichloro platinum (Ⅱ); hUCMSCs, human umbilical cord-derived mesenchymal stem cells; EVs, extracellular vesicles; ns, not significant.

In the current study, we observed a significant reduction in the number of follicles of different developmental stages in the ovaries of the CDDP-induced POI mice (***Fig. 3***). Consistent with irrevocable weight loss and ovary atrophy, hUCMSC or hUCMSC-EVs transplantation did not relieve the follicle loss at any developmental stage in the high-dose CDDP-induced POI mice (***Fig. 3A*** and ***3B***). However, treatment with hUCMSC or hUCMSC-EV significantly augmented normal follicles in the moderate-dose CDDP-induced POI mice (***Fig. 3C***). Further analysis showed that consecutive hUCMSC or hUCMSC-EV treatment significantly increased the number of healthy follicles at all developmental stages (primordial, primary, secondary, and mature follicles) in the moderate-dose CDDP-induced POI mice on the 28th day (***Fig. 3C*** and ***3D***). Thus, a moderate dose (1.5 mg/kg body weight) of CDDP was used to establish the chemotherapy-induced POI mouse model in further investigations.

**Figure 4:**
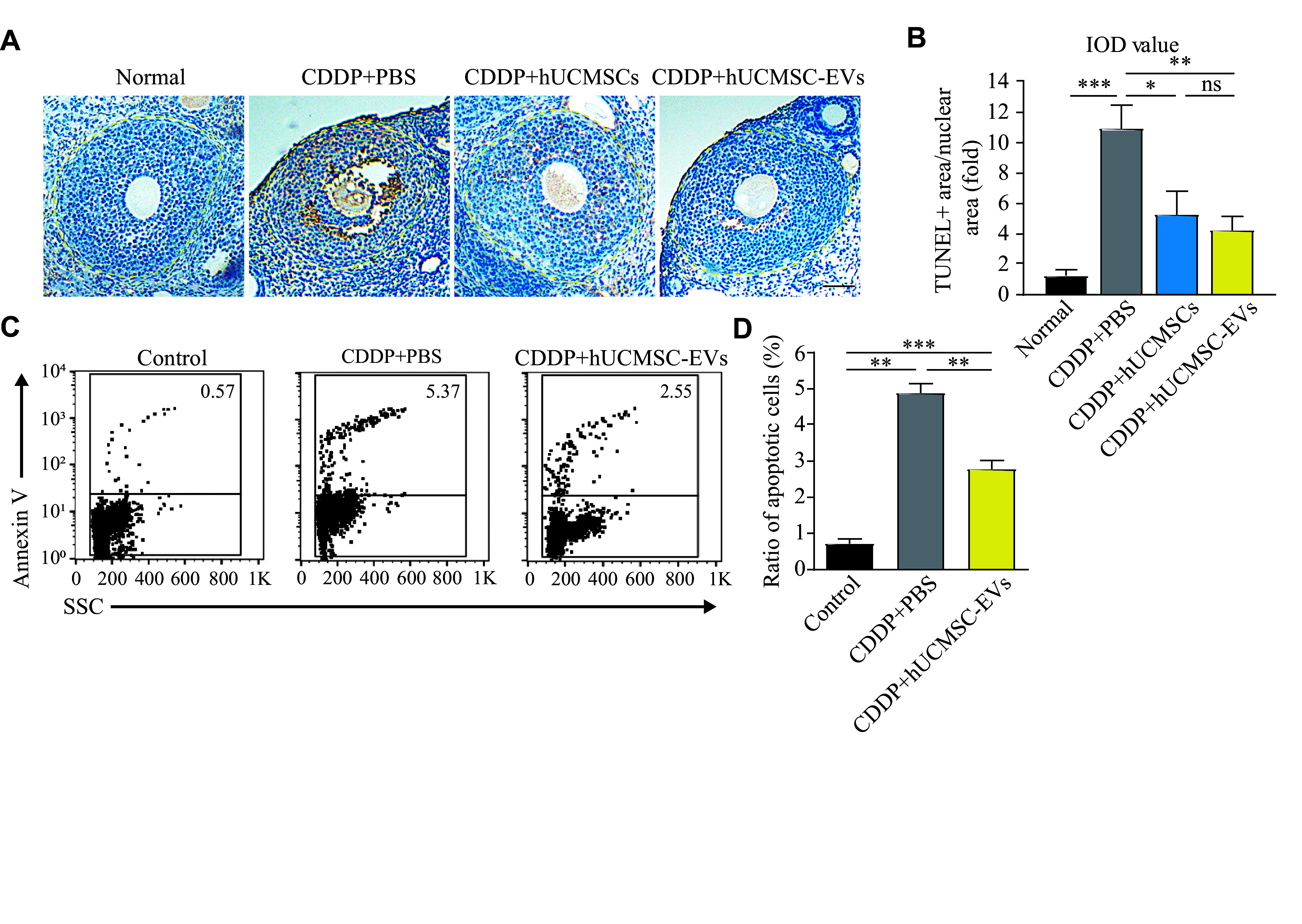
hUCMSC-EVs inhibited chemotherapy-induced apoptosis of ovarian granulosa cells (GCs) *in vivo* and *in vitro*. Mice were intraperitoneally injected with CDDP (1.5 mg/kg body weight) for seven consecutive days, followed by an administration of 2 × 10^6^ hUCMSCs or 200 μg hUCMSC-EVs for 21 days twice a week (*n* = 4 for each group). Human GC line KGN was treated with 4 μg/mL CDDP for 24 h, followed by an administration of 100 μg/mL hUCMSC-EVs for another 24 h. A: Immunohistochemical staining for TUNEL-positive GCs in the ovaries of the premature ovarian insufficiency mice treated with or without hUCMSCs and hUCMSC-EVs on the 28th day. Scale bar, 50 μm. B: The ratio of the integral optical density value of TUNEL-positive areas to that of nuclear areas (*n* = 3). C and D: Flow cytometry analysis of Annexin V-stained cells to assess apoptosis in control or CDDP-treated KGN cells followed with or without hUCMSC-EVs treatment (*n* = 3). Data are presented as mean ± standard error of the mean. Statistical analyses were performed by one-way ANOVA followed by a Bonferroni post hoc test for four-group or three-group comparisons. ^*^P < 0.05, ^**^P < 0.01, and ^***^P < 0.001. Abbreviations: CDDP, *cis*-diammine dichloro platinum (Ⅱ); hUCMSCs, human umbilical cord-derived mesenchymal stem cells; EVs, extracellular vesicles; IOD, integral optical density; SSC, side scatter; ns, not significant.

Mammalian ovarian follicular development and atresia are closely regulated by endocrine hormones and intraovarian regulators, and the CDDP-induced GC loss is a significant cause of POI^[19–20]^. Therefore, we evaluated the effects of hUCMSCs and hUCMSC-EVs on GC apoptosis in ovarian tissues of the CDDP-induced POI mice by TUNEL staining. TUNEL-positive signals in the GC areas in ovarian follicles of CDDP-induced POI mice were significantly stronger than those in normal mice, and treatment with either hUCMSC or hUCMSC-EV led to a significant reduction in these signals, indicating a decrease in GC apoptosis (***Fig. 4A*** and ***4B***). Additionally, there was no significant difference in the efficacy between hUCMSC and hUCMSC-EV treatments in reducing TUNEL-positive signals, thereby alleviating GC apoptosis (***Fig. 4A*** and ***4B***).

To further evaluate the anti-apoptotic effect of hUCMSC-EVs on the human GC line KGN, we constructed a CDDP-stimulated KGN model using 4 pg/L CDDP to induce apoptosis of KGNs based on a previous study^[22]^. The results of flow cytometry analysis showed that hUCMSC-EVs significantly relieved the CDDP-induced apoptosis in KGN cells (***Fig. 4C*** and ***4D***). In conclusion, both hUCMSCs and hUCMSC-EVs inhibited GC apoptosis *in vivo* to improve ovarian function in POI mice, and hUCMSC-EVs inhibited the apoptosis of the human GC line KGN effectively *in vitro.*

### hUCMSC-EVs transferred anti-apoptotic miRNAs to GCs in the CDDP-induced POI mice

The biological functions of EVs are largely dependent on the small noncoding RNAs they contain^[23]^. MicroRNAs (miRNAs) are among the most abundant RNAs packaged in EVs^[14]^, and certain miRNAs are involved in the inhibition of GC apoptosis^[22]^. Thus, we hypothesized that hUCMSC-EVs might alleviate cell apoptosis by transferring anti-apoptotic miRNAs to ovarian GCs.

**Figure 5:**
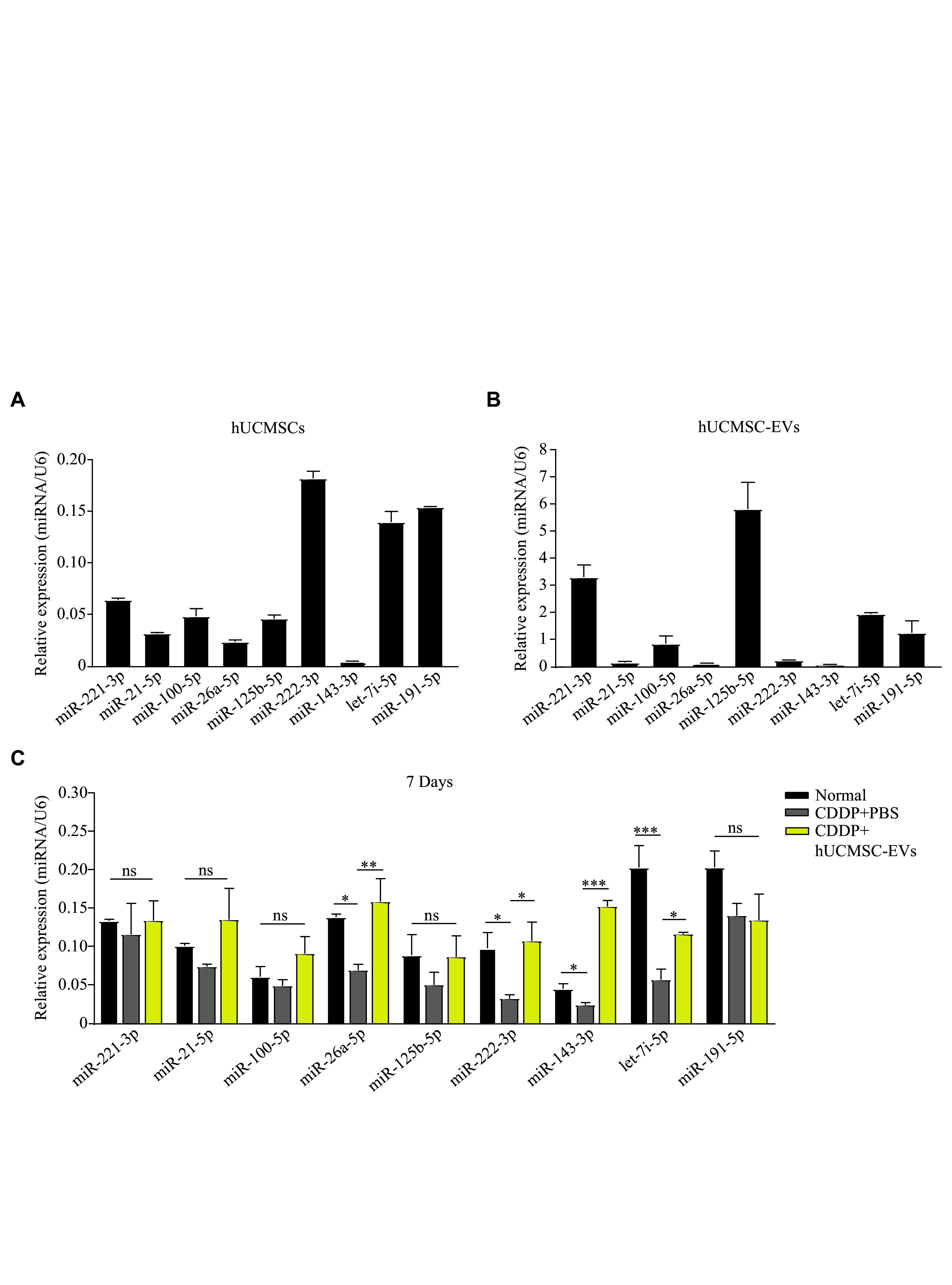
The transplantation of hUCMSC-EVs transferred anti-apoptotic miRNAs to the granulosa cells (GCs) of the CDDP-induced POI mice. A and B: The nine anti-apoptotic miRNAs that were abundant in hUCMSC-EVs were further investigated in hUCMSCs (A) and hUCMSC-EVs (B) by real-time reverse transcription-PCR. C: Mice were intraperitoneally injected with CDDP (1.5 mg/kg body weight) for seven consecutive days, followed by an administration of 200 μg hUCMSC-EVs for 7 days twice a week (*n* = 3 for each group). Real-time reverse transcription-PCR detected the indicated miRNAs in ovarian GCs of the CDDP-induced premature ovarian insufficiency mice treated with or without hUCMSC-EVs on the 14th day. Data are presented as mean ± standard error of the mean. The expression levels of the miRNAs were normalized to the U6 snRNA. Statistical analyses were performed by one-way ANOVA followed by a Bonferroni post hoc test for three-group comparisons. ^*^P < 0.05, ^**^P < 0.01, and ^***^P < 0.001. Abbreviations: CDDP, *cis*-diammine dichloro platinum (Ⅱ); hUCMSCs, human umbilical cord-derived mesenchymal stem cells; EVs, extracellular vesicles; miR, miRNA; U6, U6 small nuclear RNA; ns, not significant.

According to the published hUCMSC-EV miRNA-seq analyses, we listed the top 10 most abundant miRNAs from each of the five studies to identify the shared miRNAs across these investigations^[24–28]^. Nine of these miRNAs in hUCMSC-EVs have been reported to exhibit anti-apoptotic properties, *i.e.*, miR-100-5p, miR-21-5p, miR-143-3p, let-7i-5p, miR-125b-5p, miR-26a-5p, miR-221-3p, miR-191-5p, and miR-222-3p (***Fig. 5A***). Interestingly, we observed different expression patterns of these nine miRNAs between hUCMSCs and hUCMSC-EVs, and the highly expressed miRNAs in hUCMSCs were not necessarily abundant in hUCMSC-EVs (***Fig. 5A*** and ***5B***). Among these nine miRNAs, the expression levels of miR-26a-5p, miR-222-3p, miR-143-3p, and let-7i-5p were significantly decreased in ovarian GCs of the CDDP-induced POI mice, and this downregulation was reversed by the treatment of hUCMSC-EVs (***Fig. 5C***). These results indicate that the four miRNAs (*i.e.*, miR-26a-5p, miR-222-3p, miR-143-3p, and let-7i-5p) may inhibit the CDDP-induced GC apoptosis.

### hUCMSC-EVs downregulated pro-apoptotic gene expression levels in GCs of the CDDP-induced POI mice

**Figure 6:**
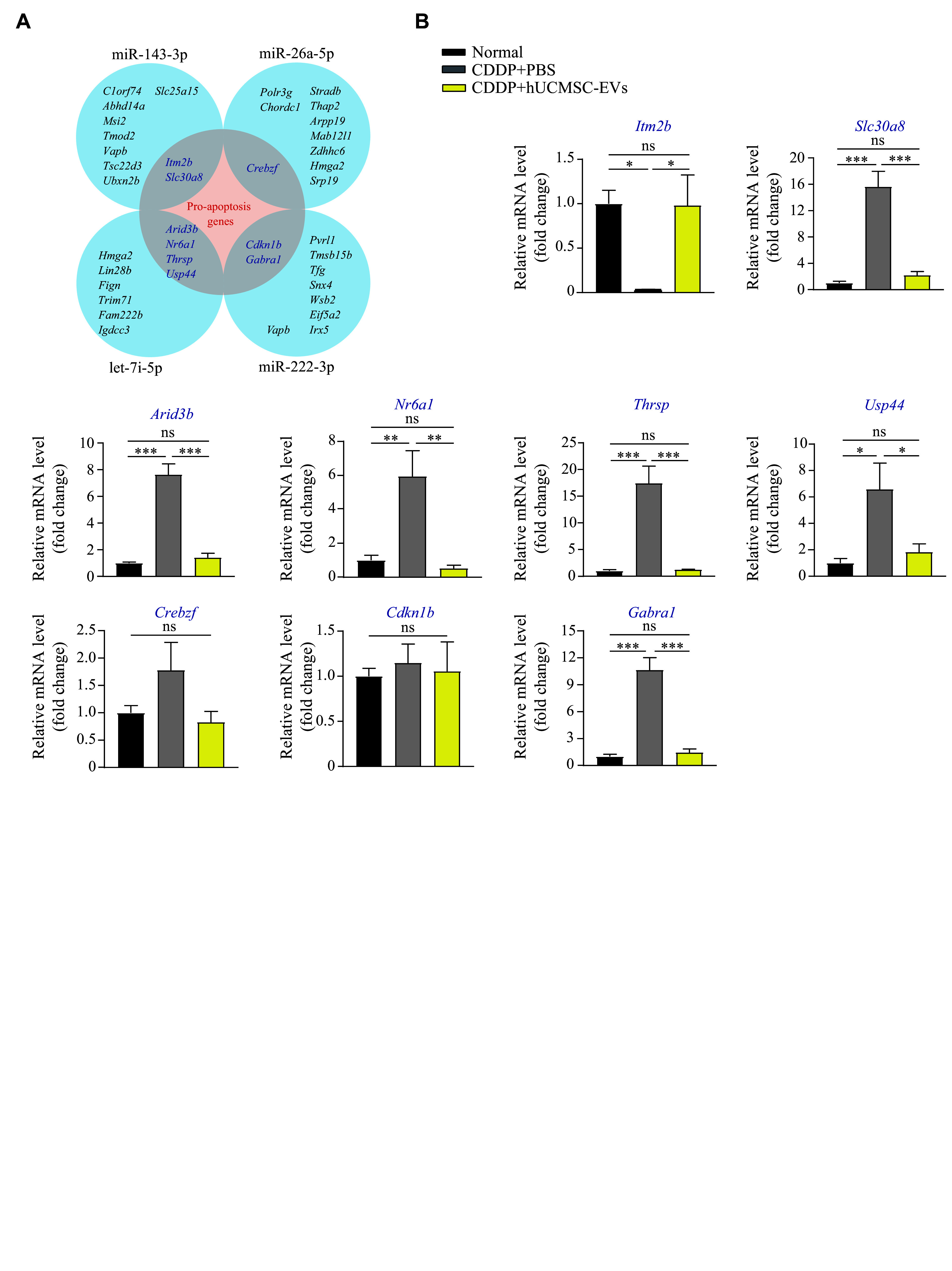
Supplementary anti-apoptotic miRNAs functioned by downregulating pro-apoptotic gene mRNAs in granulosa cells (GCs). A: Venn diagram showing the pro-apoptotic genes included in four differential miRNA substrate genes that were predicted by TargetScan. B: Mice were intraperitoneally injected with CDDP (1.5 mg/kg body weight) for seven consecutive days, followed by an administration of 200 μg hUCMSC-EVs for 7 days twice a week (*n* = 3 each group). The pro-apoptotic gene shown in panel A was investigated in ovarian GCs of the CDDP-induced premature ovarian insufficiency mice treated with or without hUCMSC-EVs on the 14th day. Statistical analyses were performed by one-way ANOVA followed by a Bonferroni post hoc test for three-group comparisons. Data are presented as mean ± standard error of the mean. ^*^P < 0.05, ^**^P < 0.01, and ^***^P < 0.001. Abbreviations: CDDP, *cis*-diammine dichloro platinum (Ⅱ); hUCMSC-EVs, human umbilical cord-derived mesenchymal stem cell-derived extracellular vesicles; ns, not significant.

**Figure 7:**
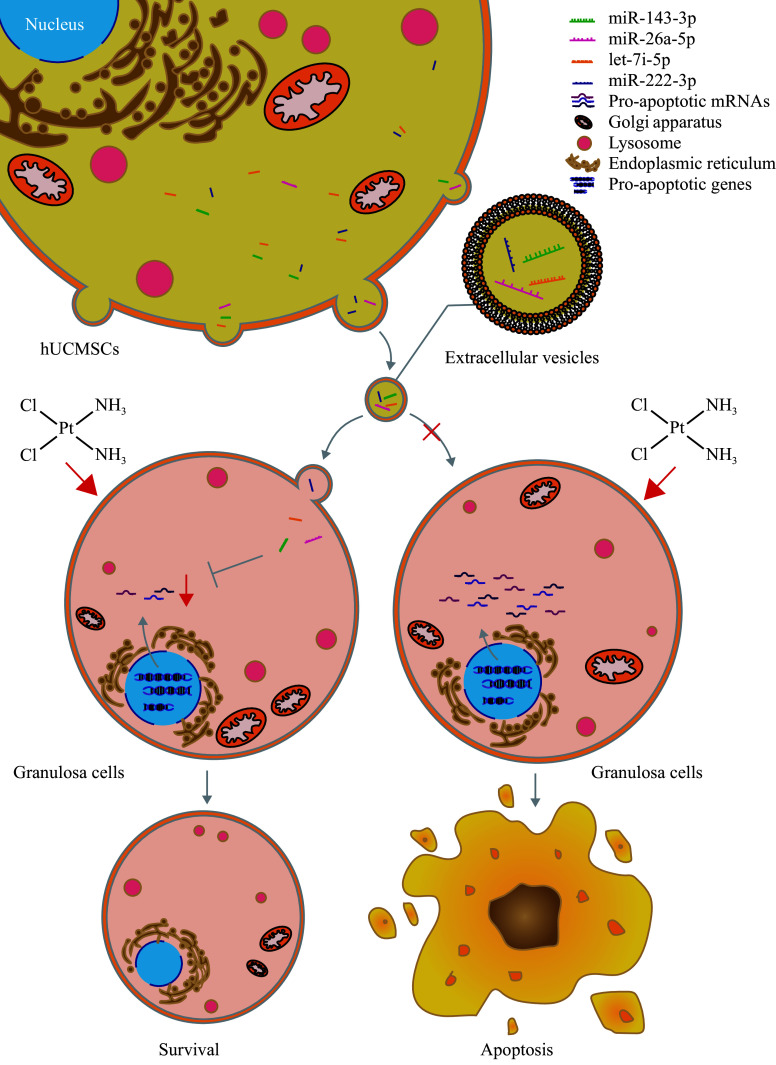
A proposed mechanism by which hUCMSC-EVs mitigate GC apoptosis in the CDDP-induced POI mice. In the *cis*-diammine dichloro platinum (Ⅱ)-induced premature ovarian insufficiency mice, anti-apoptotic miRNAs delivered by human umbilical cord-derived mesenchymal stem cell-derived extracellular vesicles (hUCMSC-EVs) down-regulate mRNA levels of the pro-apoptotic genes in ovarian granulosa cells, thereby leading to the reduced granulosa cell apoptosis and better ovarian function.

Functioning as crucial post-transcriptional regulators of gene expression, miRNAs are anticipated to govern the activity of over 60% of all protein-coding genes in mammals. miRNAs may function post-transcriptionally, usually by base-pairing to the mRNA 3′ UTR to repress translation, and lead to mRNA decay^[29]^. We suspected that miR-26a-5p, miR-222-3p, miR-143-3p, and let-7i-5p might downregulate pro-apoptotic gene mRNAs, thereby reducing the synthesis of pro-apoptotic proteins through conventional mechanisms. Therefore, we predicted some potential pro-apoptotic substrates of these four miRNAs on TargetScan, in which a higher predictive score means a greater possibility of binding to a specific miRNA. We identified the top 10 highest-scored substrate mRNAs for each miRNA, and selected nine pro-apoptotic gene mRNAs from these candidates (***Fig. 6A***). Then, we detected the expression levels of these nine mRNAs in the ovarian GCs from normal mice and the moderate-dose CDDP-induced POI mice with or without hUCMSC-EV treatment, and found that the mRNA levels of most of these pro-apoptotic target genes, including *Slc30a8*, *Arid3b*, *Nr6a1*, *Thrsp*, *Usp44*, and *Gabra1*, were significantly increased after CDDP injection but downregulated after hUCMSC-EV treatment (***Fig. 6B***). Only *Crebzf* and *Cdkn1b* showed no significant change in mRNA levels after CDDP injection or hUCMSC-EV treatment (***Fig. 6B***). As demonstrated in ***Fig. 7***, miRNAs transferred by hUCMSC-EVs may downregulate multiple pro-apoptotic mRNAs, leading to the reduced GC apoptosis in the POI mice.

## Discussion

With the development of oncotherapy, chemotherapeutic drugs have been widely used to treat cancer patients, inevitably leading to an increasing incidence of the chemotherapy-induced POI cases. Currently, the therapies used to treat or relieve POI still have many side effects. For example, hormone replacement therapy, which aims at replicating normal ovarian hormone production, may potentially increase the risk of gynecological oncology recurrence. The transmission of hormones through breast milk has been reported to cause jaundice or breast enlargement in neonates^[30]^. Additionally, hormone replacement therapy may not fundamentally treat the chemotherapy-induced POI, as it fails to rescue GC apoptosis following chemotherapy. Although embryo or oocyte cryopreservation is the current standard strategy for preserving the fertility of POI patients^[31]^, it does not alleviate symptoms associated with ovarian hormone deficiency. Therefore, safe and effective therapies are urgently needed to restore ovarian function after chemotherapy.

In clinics, the therapeutic effects of CDDP depend heavily on the dosage, while the chemotherapy doses and regimens depend on cancer types. For example, a moderate dose of CDDP (50 mg/m^2^ intravenously every three weeks, equal to 1.33 mg/kg for a person weighing 60 kg) is usually applied for ovarian cancer oncotherapy. A high dose of CDDP (75 to 100 mg/m^2^ intravenously every three to four weeks, equal to 2.00 to 2.67 mg/kg for a person weighing 60 kg) is usually applied for esophageal cancer, Hodgkin's lymphoma, non-Hodgkin's lymphoma, osteosarcoma, or lung cancer treatment^[32]^. Therefore, it is crucial to adopt dose-based precision medication in chemotherapy according to the type of cancer. Additionally, many precise drug delivery methods reduce CDDP dosage without weakening its effect in cancer treatment^[33–34]^. Considering the fertility preservation of female patients, a moderate-dose chemotherapy regimen or precise CDDP delivery method accompanied by hUCMSC-EVs transplantation may be a feasible therapy in the future.

Stem cell-based therapies have been reported to inhibit GC apoptosis and follicular atresia^[35]^. In the current study, we focused on the effectiveness of relieving the CDDP-induced POI with hUCMSC-EVs instead of hUCMSCs both *in vitro* and *in vivo*, because hUCMSC-EVs are more stable and safer for clinical application. We successfully isolated and characterized hUCMSC-EVs and found that these vesicles specifically targeted ovaries injured by CDDP. We also observed that hUCMSC-EVs had similar protective or restorative effects on ovarian structure and follicle numbers by inhibiting GC apoptosis in the POI mouse model.

Several miRNAs confer resistance to CDDP in cells, and these miRNAs are typically delivered by extracellular vesicles or exosomes^[36]^. One significant mechanism of these miRNAs is the downregulation of pro-apoptotic mRNAs and intracellular signaling pathways^[37]^. Therefore, miRNAs transferred by hUCMSC-EVs may help GCs survive CDDP treatment by degrading pro-apoptotic mRNA or reducing their translation activity. In the current study, we found that abundant miRNAs might be transferred to ovarian GCs, thereby degrading the mRNAs of several pro-apoptotic genes. This may represent an important molecular mechanism underlying the anti-apoptotic effect of hUCMSC-EVs.

In the current study, we confirmed that hUCMSC-EVs alleviated the CDDP-induced ovarian GC apoptosis *in vivo*. Subsequently, we aimed to validate the potential protective effect of hUCMSC-EVs on the CDDP-induced apoptosis in human ovarian GCs. Because of lacking access to primary human GCs or the inability to perform *in vivo* experiments in humans, we used a human KGN GC line instead. Similarly, we measured the levels of anti-apoptotic miRNAs in GCs isolated from mouse ovaries after transplanting hUCMSC-EVs for seven days. This approach was adopted as direct transplantation of hUCMSC-EVs into POI patients to investigate the anti-apoptotic mechanism of hUCMSC-EVs is not feasible.

Furthermore, we observed that the expression of *Itm2b* was significantly suppressed in the chemotherapy mouse model, which was not consistent with other pro-apoptotic genes examined in the current study. Other investigators suggested that the expression level of *Itm2b* increased 72 h after CDDP treatment^[38]^, and miR-143-3p inhibited the expression level of *Itm2b* to promote cell proliferation^[39]^. The discrepancies in these findings may be attributed to differences in cell types, CDDP dosage, and CDDP treatment duration in the current study. Additionally, ITM2B is a ubiquitously expressed membrane protein, acts as a receptor or part of a receptor complex, and participates in signal transduction pathways in human neuronal cells, thereby promoting capacitation in spermatozoa, and modulating the homeostasis of metabolites like uric acid in proximal tubule cells^[40]^. The specific role of ITM2B in ovarian GCs has not been reported since then. ITM2B may exhibit other physiological functions in ovarian GCs, which are more significant than its pro-apoptotic effect. In addition, compared with miR-143-3p, there may be other mechanisms regulating the mRNA level of *Itm2b* in GCs.

While acknowledging the significance of the current study, it is important to note certain limitations. In this study, we only observed ovary morphology, weight, and follicle number at all stages. However, how these alterations in ovaries may affect the fertility of mice requires further investigation. Additionally, we focused exclusively on the mRNA levels of pro-apoptotic genes after transplanting hUCMSC-EVs into the CDDP-induced POI mice. It is essential to explore the downstream mechanisms that inhibit ovarian GC apoptosis to elucidate the effects of other mRNAs and proteins on GC apoptosis in future research.

In conclusion, we demonstrated that hUCMSC-EVs, similar to hUCMSCs, inhibited ovarian GC apoptosis and relieved the CDDP-induced ovarian dysfunction in mice with the moderate-dose CDDP-induced POI. Additionally, we identified four miRNAs that might be delivered by hUCMSC-EVs and involved in downregulating pro-apoptotic gene mRNAs in ovarian GCs, thereby reducing GC apoptosis and improving ovarian functions. The findings of the current study may provide valuable insights into the development of effective therapies with the minimal side effects for adolescent and young adult female cancer patients.
